# *Amycolatopsis camponoti* sp. nov., new tetracenomycin-producing actinomycete isolated from carpenter ant *Camponotus vagus*

**DOI:** 10.1007/s10482-022-01716-w

**Published:** 2022-02-26

**Authors:** Yuliya V. Zakalyukina, Ilya A. Osterman, Jacqueline Wolf, Meina Neumann-Schaal, Imen Nouioui, Mikhail V. Biryukov

**Affiliations:** 1 Scientific Center of Genetics and Life Sciences, Sirius University of Science and Technology, Sochi, Russia 354340; 2 Department of Soil Science, Lomonosov Moscow State University, Moscow, Russia 119991; 3 Skolkovo Institute of Science and Technology, Skolkovo, Moscow Region Russia 143025; 4 Department of Chemistry and A.N. Belozersky Institute of Physico-Chemical Biology, Lomonosov Moscow State University, Moscow, Russia 119991; 5 Leibniz Institute DSMZ–German Collection of Microorganisms and Cell Cultures, 38124 Braunschweig, Germany; 6 Department of Biology, Lomonosov Moscow State University, Moscow, Russia 119991

**Keywords:** *Amycolatopsis camponoti* sp. nov., Actinobacteria, *Amycolatpsis* sp, Tetracenomycin X, *Camponotus vagus*

## Abstract

An actinobacterial strain A23^T^, isolated from adult ant *Camponotus vagus* collected in Ryazan region (Russia) and established as tetracenomycin X producer, was subjected to a polyphasic taxonomic study. Morphological characteristics of this strain included well-branched substrate mycelium and aerial hyphae fragmented into rod-shaped elements. Phylogenetic analyses based on 16S rRNA gene and genome sequences showed that strain A23^T^ was most closely related to *Amycolatopsis pretoriensis* DSM 44654^T^. Average nucleotide identity and digital DNA–DNA hybridization values between the genome sequences of isolate A23^T^ and its closest relative, *Amycolatopsis pretoriensis* DSM 44654^T^, were 39.5% and 88.6%, which were below the 70% and 95–96% cut-off point recommended for bacterial species demarcation, respectively. The genome size of the isolate A23^T^ was 10,560,374 bp with a DNA G + C content of 71.2%. The whole-cell hydrolysate contained *meso*-diaminopimelic acid and arabinose and galactose as main diagnostic sugars as well as ribose and rhamnose. It contained MK-9(H4) as the predominant menaquinone and *iso*-C_16:0_, *iso*-C_15:0_, *anteiso*-C_17:0_ and C_16:0_ as the major cellular fatty acids. Diphosphatidylglycerol and phosphatidylethanolamine prevailed among phospholipids. Mycolic acids were not detected. Based on the phenotypic, genomic and phylogenetic data, isolate A23^T^ represents a novel species of the genus *Amycolatopsis*, for which the name *Amycolatopsis camponoti* sp. nov. is proposed, and the type strain is A23^T^ (= DSM 111725^T^ = VKM 2882^T^).

**Supplementary Information:**

The online version contains supplementary material available at 10.1007/s10482-022-01716-w.

## Introduction

The genus *Amycolatopsis* Lechevalier et al. 1986 belonged to the family *Pseudonocardiaceae* Embley et al. 1989 (order *Pseudonocardiales* Labeda and Goodfellow 2015, class *Actinomycetia* Salam *et al.* 2020 (Salam et al. 2020), phylum *Actinomycetota* corrig. Goodfellow 2021 (Oren and Garrity 2021)), and encompasses 85 validly published species names with *Amycolatopsis orientalis* as its type species (https://lpsn.dsmz.de/genus/amycolatopsis). *Amycolatopsis* is aerobic to facultatively anaerobic and characterized by branched vegetative hyphae that undergo fragmentation into rod-like and squarish elements. Whole-cell hydrolysates are rich in *meso*-2,6-diaminopimelic acid, along with arabinose and galactose as whole-cell sugars. The peptidoglycan is of the A1g type. Muramic acid moieties are N-acetylated. Does not contain mycolic acids. The diagnostic phospholipid is phosphatidylethanolamine and/or phosphatidylmethylethanolamine while the occurrence of diphosphatidylglycerol, phosphatidylglycerol, phosphatidylinositol and phosphatidylinositol mannosides is variable (Tan and Goodfellow 2015). *Amycolatopsis* have been described from diverse environments such as soil, vegetation, human and animal clinical sources, fresh water, rock and subterranean sites (Lee 2009).

The 16S rRNA gene phylogeny provides a useful framework for inferring the relationships between genera in the *Pseudonocardiaceae* family, but did not seem to have enough taxonomic resolution to distinguish species. The use of multi-locus sequence analysis (MLSA) is able to clarify the taxonomic positions of *Amycolatopsis* species (Glaeser and Kämpfer 2015). The most reliable way to identify actinobacteria strains is the comparison of genomes from the point of view of DNA–DNA relatedness and construction whole-genome phylogenetic trees (Nouioui et al. 2018).

The studies of 66 publicly available representative genome sequences of *Amycolatopsis* type strains (https://www.ncbi.nlm.nih.gov/genome/browse#!/prokaryotes/Amycolatopsis) have revealed that *Amycolatopsis* have comparatively large genomes from nearly 5.62 Mb (*Amycolatopsis granulosa* DSM 45669^T^) to 10.94 Mb (*Amycolatopsis anabasis* EGI 650086^T^), GC content from 67.8% (*Amycolatopsis palatopharingis* DSM 44832^T^) to 72.7% (*Amycolatopsis arida* DSM 45648^T^), while median genome size and DNA G + C content are 9.08 Mb and 70.1%, respectively. The circular chromosomes containing over 20 secondary metabolic gene clusters (Kumari et al. 2016).

The genus *Amycolatopsis* comprises a large group of commercially and medically important actinobacteria capable of producing two major types of antibiotics: glycopeptides and polyketides (Kisil et al. 2021). In this paper, we describe strain A23^T^, isolated from body of ant *Camponotus vagus* collected in Ryazan region, Russia. This strain is able to produce the aromatic polyketide antibiotic tetracenomycin X (TcmX) and its new congener 6-hydroxytetracenomycin X (6-OH-Tcm X) which possess antimicrobial and cytotoxic activity (Osterman et al. 2020; Alferova et al. 2021). As recently demonstrated TcmX and 6-OH-Tcm X are potent inhibitors of protein synthesis due to the fact that they bind to the large subunits of prokaryotic or eukaryotic ribosomes, within the polypeptide exit tunnel (Osterman et al. 2020).

Polyphasic study was used to clarify the taxonomic position of novel strain A23^T^. We propose to establish this strain as a representative for the novel species of the genus *Amycolatopsis*, with the name *Amycolatopsis camponoti* sp. nov.

## Materials and methods

### Collection and microbial isolation

Strain A23^T^ was isolated from bodies of adult workers of *Camponotus vagus* collected in Kasimovsky District, Ryazan region, Russia (55.01138 N, 41.73078 E) (Zakalyukina et al. 2021). Five individuals were washed three times in sterile distilled water and then crushed by tissue microhomogenizer with sterile saline solution. Aliquots of this mixtures were spread over M490 medium (HiMediaLab) supplemented with nystatin and nalidixic acid at final concentrations of 250 μg/mL and 10 μg/mL, respectively, and incubated for 14 days at 28 °C (Zakalyukina et al. 2019). The strain was purified and maintained on Organic medium 79 (Prauser and Falta 1968), and preserved as suspension of mycelial fragments and spores in 20% (v/v) glycerol at − 20 °C.

### Genome features and phylogenomic analysis

Genome of strain A23^T^ was sequenced de novo by Novogene Co., Ltd. (https://en.novogene.com/), using the Illumina NovaSeq 6000 platform and fully annotated using RAST prokaryotic genome annotation service (https://rast.nmpdr.org/) and submitted in GenBank (assembly accession: GCA_902497555.1) (Osterman et al. 2020).

Average nucleotide identity (ANI) (Rodríguez-R and Konstantinidis 2016) and in silico digital DNA:DNA hybridization (DDH) were calculated using JSpecies WS (http://jspecies.ribohost.com/jspeciesws/), and GGDC method, with the recommended formula 2, available at the TYGS web service, respectively (Meier-Kolthoff and Göker 2019).

Phylogenomic analysis was performed using Type (Strain) Genome Server (https://tygs.dsmz.de/). The phylogenomic tree inferred with FastME 2.1.6.1 (Lefort et al. 2015) from GBDP distances calculated from genome sequences. The branch lengths are scaled in terms of GBDP distance formula d5.

### 16S rRNA phylogeny

The full-length 16S rRNA gene sequences of strain A23^T^ was extracted from the whole genome sequence (CABVGP010000001.1) and was compared to sequences of type strains in the EzBioCloud database (www.ezbiocloud.net).

Evolutionary trees based on 16S rRNA gene sequences were inferred with the neighbour-joining, maximum-parsimony and maximum-likelihood tree-making algorithms after CLUSTAL W alignment by using MEGA software version X (Kumar et al. 2018) (https://www.megasoftware.net). These evolutionary analyzes involved 24 nucleotide sequences. All positions with less than 95% site coverage were eliminated, i.e., fewer than 5% alignment gaps, missing data, and ambiguous bases were allowed at any position (partial deletion option). There were a total of 1414 positions in the final dataset.

### Phenotypic characterization

Cultural characteristics of strain A23^T^ were observed on the range of ISP 2-ISP 7 media (Shirling and Gottlieb 1966), Organic medium 79 and modified Bennett’s agar (Tan et al. 2006b) after cultivation up to 14 days at 28 °C. Cell morphology of strain A23^T^ on Organic medium 79 after cultivation at 28 °C for 10 days was studied using scanning electron microscopy (JSM-6380LA, JEOL). Motility test was carried out using the “hanging drop” method by light microscope (Fisherbrand AX-502, Thermo Fisher Scientific).

Carbon source utilization was assessed on basal medium ISP 9 (Shirling and Gottlieb 1966) with addition of 0.04% solution of bromocresol purple at 28 °C for 14 days. Enzyme activities were estimated using paper indicator system (NPO Microgen, Russia) according to the manufacturer’s recommendations at 28 °C for 7 days. The degradation of casein, starch and cellulose was estimated on clearing of the insoluble compounds around areas of growth (Williams et al. 1983). The ability of strain to grow at a different pH (pH 5.0, pH 6.0, pH 7.0, pH 8.0, pH 9.0) was tested on a modified ISP 9 medium (g/L: (NH_4_)_2_SO_4_–2.64, MgSO_4_ × 7H_2_O–1, glucose—10, agar—20), buffered with phosphate solutions according to Williams et al. (Williams et al. 1971), at 28 °C for 14 days. The growth at different range of temperature (5 °C, 10 °C, 20 °C, 30 °C, 40 °C) and salinity (1%, 5%, 8%) were assessed on Organic medium 79 after 14 days of incubation.

### Chemotaxonomy

Cell biomass of strains A23^T^ and *A*. *pretoriensis* DSM 44654^T^ were obtained from cultures grown in DSMZ 554 broth medium and on a rotary shaker (180 r.p.m) at 28 °C. After 120-h growth cells were centrifuged, washed three times in sterile distilled water and freeze-dried. Whole-cell sugars (Lechevalier and Lechevalier 1970; Staneck and Roberts 1974), menaquinone (Collins et al. 1985) and diaminopimelic acids analyses (Schleifer and Kandler 1972) were carried out. Standard chromatographic procedures were used to determine polar lipid pattern for strain A23^T^ and *A. pretoriensis* DSM 44654^T^ following the protocol of Minnikin et al. (1984).

Cellular fatty acids extracts were prepared using minor modifications of the protocol of Miller (Miller 1982) and Kuykendall et al. (Kuykendall et al. 1988). Gas chromatography (Agilent 6890N instrument) was used to analyse the fatty acid methyl esters which were identified using Sherlock Microbial Identification system (MIDI, Microbial ID, Newark, DE 19711 U.S.A.) and the Actin6 database (Sasser 1990). The analysis was supplemented by a GC-MS run on an Agilent GC-MS 7000D for identity confirmation. Mycolic acids extraction was performed according to Vilchéze and Jacobs (Vilcheze and Jacobs 2007). Cell lysis was carried out in KOH/MeOH solution at 95 °C overnight and extracted with chloroform. Dried extracts were recovered in chloroform:MeOH (9:1) and analyzed in negative ion mode on an Agilent QTof mass spectrometer by direct infusion into the ESI source (300 µL/min). Mycolic acids were identified by comparison of the exact masses of known mycolic acid structures to the measured ones (Bouam et al. 2018).

### Analysis of bioactive compound biosynthetic gene clusters and potential pathogenicity

Secondary metabolite biosynthetic gene clusters in complete genome strain A23^T^ (CABVGP010000000) and *A. pretoriensis* DSM 44654^T^ (GenBank Accessions FNUJ01000000) were identified with the bacterial version of antiSMASH 6.1.0 (https://antismash.secondarymetabolites.org/). Homologous regions on each genome were identified using NCBI Blastn (https://blast.ncbi.nlm.nih).

For predicting the pathogenicity of A23^T^ and closely related strains we used *PathogenFinder* (http://cge.cbs.dtu.dk/services/PathogenFinder/), a web-server for the prediction of bacterial pathogenicity by analysing the input genome (Cosentino et al. 2013).

## Results and discussion

*Amycolatopsis* strains are well known as producers for the commercially used antibiotics, e.g. vancomycin, rifamycin, eremomycin (Kisil et al. 2021). Other compounds with antibacterial, antifungal or antiviral properties that have been derived from *Amycolatopsis* strains are quartromycin, octacosamicin, chelocardin, kigamicin and the macrotermycins A–D (Chen et al. 2016; Kumari et al. 2016; Beemelmanns et al. 2017). It has been shown that there is a relationship between the distribution of biosynthetic gene clusters (BGCs) and phylogenetic lineages in *Amycolatopsis* genomes. *Amycolatopsis* strains that produce, or have the potential to produce, a particular class of antibiotic are phylogenetically related (Adamek et al. 2018). Strain A23^T^ was previously known to produce bioactive compounds (Osterman et al. 2020; Alferova et al. 2021) was subjected to a polyphasic taxonomic approach to clarify its taxonomic position.

**Fig. 1:**
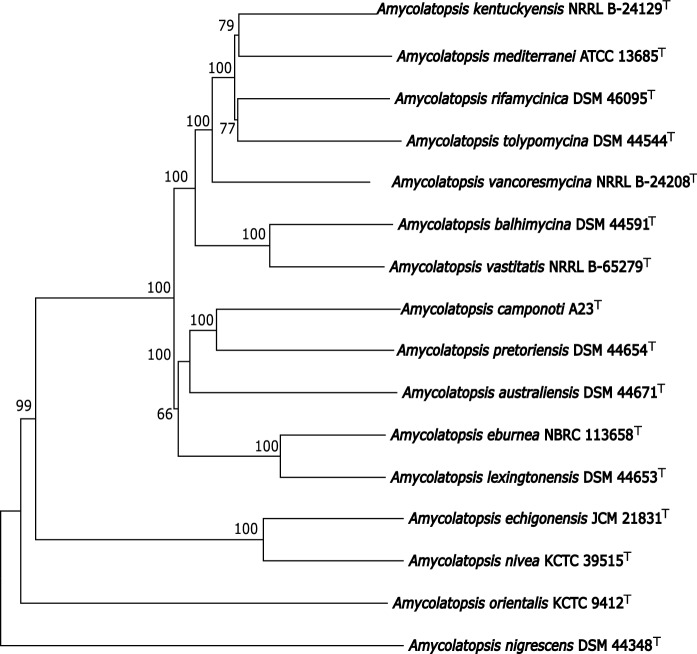
Phylogenetic tree based on whole-genome sequences from A23^T^ and 15 *Amycolatopsi*s type strains. Numbers above branches are GBDP pseudo-bootstrap support values > 60% from 100 replications, with an average branch support of 93.9%. The tree was rooted at the midpoint

Phylogenomic analysis based on whole-genome sequences showed that strain A23^T^ formed a well-supported monophyletic clade with *A. pretoriensis* DSM 44654^T^ with 100% bootstrap value (Fig. 1). The neighbor-joining (Fig. S1), maximum-likelihood (Fig. S2) and maximum-parsimony (Fig. S3) trees based on 16S rRNA full-length gene sequences revealed that strain A23^T^ formed a subclade in the *Amycolatopsis* tree together with *Amycolatopsis pretoriensis* DSM 44654^T^ and *Amycolatopsis lexingtonensis* NRRL B-24131^T^, concurrently the similarity detected by EzBioCloud is 99.1% and 99.2% respectively. The isolate was also found to share relatively high 16S rRNA gene similarities with the type strains of *A. rifamycinica* DSM 46095^T^ (99.3%), *A. kentuckyensis* NRRL B-24129^T^ (99.1%), *A. tolypomycina* DSM 44544^T^ (98.9%), *A. vancoresmycina* DSM 44592^T^ (98.8%), *A. eburnea* NBRC 113658^T^ (98.6%), *A. balhimycina* DSM 44591^T^ (98.5%), *A. mediterranei* NRRL B-3240^T^ (98.4%), *A. vastitatis* NRRL B-65279^T^ (98.3%) and *A. australiensis* DSM 44671^T^ (97.9%).

Previous phylogenomic analyses revealed that the *Amycolatopsis* clade is divided into four major phylogenomic subclades (Adamek et al. 2018; Sangal et al. 2018). This study showed that strain A23^T^ is closely related to type strains within the group B subclades sensu Adamek et al. 2018 and Sangal et al. 2018. The study of Sánchez-Hidalgo with colleagues (Sánchez-Hidalgo et al. 2018) showed 3 major clades and 11 groups. The strains closely related to strain A23^T^ are within subclade AOS, group C sensu Sánchez-Hidalgo et al. 2018. The genome-based phylogenies of Adamek et al. 2018 and Sánchez-Hidalgo et al. 2018 were based on MLSA, while Sangal et al. 2018 and Teo et al 2021 were based on core protein sequences/core-proteome. The phylogenomic analysis presented in this study, based on GBDP distances calculated from genome sequences, showed consistency with previous studies (Fig. 1).

The complete genome size of strain A23^T^ was 10,560,374 bp with DNA G + C content of 71.2%, which was consistent with the G + C content of the genus *Amycolatopsis* (Teo et al. 2021)*.* Similar genome features were observed for the closest neighbor *A. pretoriensis* DSM 44654^T^ (genome size 10,299,026 bp and G + C content 71.2%). Representatives of the group B are characterized by the presence of a high number of biosynthetic gene clusters ranged from 28 to 41 per genome (Adamek et al. 2018). That fact explains the ability of majority of this clade to produce antibiotics and/or bioactive molecules (Tan and Goodfellow 2015). The genome properties of strain A23^T^ and other type strains within group B sensu Adamek et al. 2018 and Sangal et al. 2018 are summarized in Table 1.

**Table 1:** General features of the genome of strain A23^T^ and its closely related species of the genus *Amycolatopsis*

Genomic features	1	2	3	4	5	6	7	8	9	10	11	12
Size (Mbp)	10,560	9312	10,858	10,230	10,184	10,536	10,246	10,299	9202	10,363	9837	10,670
Total gene	10,054	8746	9886	9513	9811	9947	9611	9886	8636	9543	8958	10,021
Contigs	6	10	10	75	949	916	–	31	88	4	82	163
G + C content (%)	71.2	71.9	70.8	71.8	71.7	71.5	71.3	71.2	71.8	71.7	72.0	70.8
No. of rRNA clusters	12	14	12	24	4	12	12	11	19	12	21	3
No. of tRNA clusters	58	53	55	51	54	53	54	52	57	52	54	56
Contig N50	3,441,384	9,115,762	2,552,073	573,040	19,188	19,914	–	828,924	210,173	8,172,406	440,492	236,110
Contig L50	2	1	2	6	158	156	–	5	17	1	8	16
No. proteins	9936	8422	9516	9339	9248	9452	9430	9807	8370	9255	8746	9686
Completeness of genome^a^, %	99.1	99.1	99.1	99.1	98.1	97.2	99.1	99.1	99.1	99.1	95.3	99.1
Quality of genome^a^, %	47.1	52.1	52.0	42.6	51.0	54.7	66.1	42.5	42.6	37.6	43.3	52.1

Strains, (GenBank assembly accessions are indicated in parentheses): 1, A23^T^ (GCA_902497555.1), 2, *A. australiensis* DSM 44671^T^ (GCA_900119165.1); 3, *A. balhimycina* DSM 44591^T^ (GCA_000384295.1); 4, *A. eburnea* NBRC 113658^T^ (GCA_003937945.1); 5, *A. kentuckyensis* NRRL B-24129^T^ (GCA_002155975.1); 6, *A. lexingtonensis* NRRL B-24131^T^ (GCA_014873755.1); 7, *A. mediterranei* ATCC 13685^T^ (GCF_000454025.1); 8, *A. pretoriensis* DSM 44654^T^ (GCA_900107925.1); 9, *A. rifamycinica* DSM 46095^T^ (GCA_000695625.1); 10, *A. tolypomycina* DSM 44544^T^ (GCA_900105945.1); 11, *A. vancoresmycina* DSM 44592^T^ (GCA_000388135.1); 12, *A. vastitatis* NRRL B-65279^T^ (GCA_002234595.1)

^a^The genome completeness and quality of A23^T^ and all closely related type strains was evaluated using a web service MiGA (http://microbial-genomes.org/)

The ANI and in silico DDH values between strain A23^T^ and strain DSM 44654^T^ were 39.5% and 88.6%, respectively. The ANI and in silico DDH values between strain A23^T^ and other related species of the genus *Amycolatopsis* were below the recommended thresholds of 95–96% and 70% for species demarcation (Ciufo et al. 2018; Meier-Kolthoff and Göker 2019) (Table S1).

**Fig. 2:**
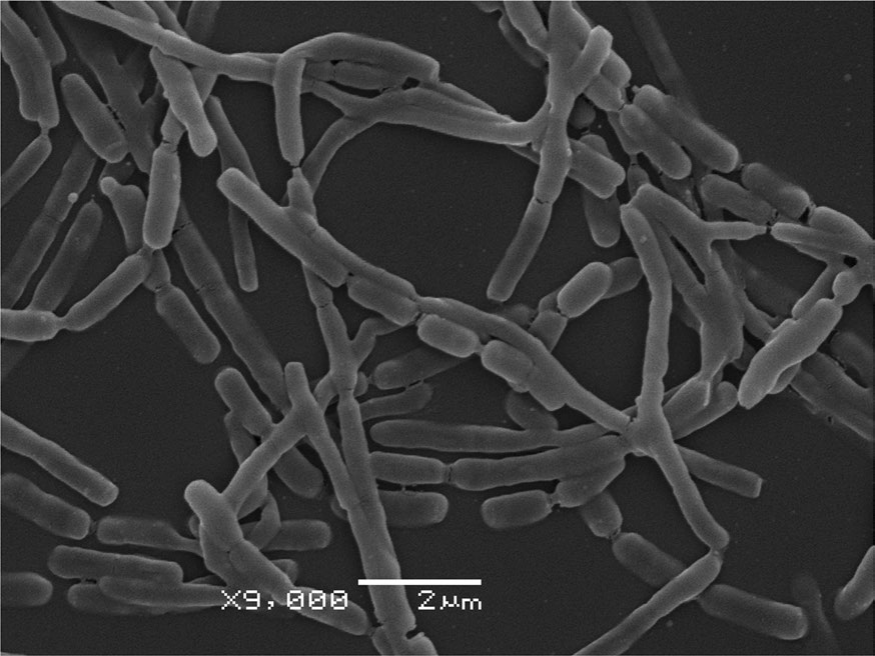
Scanning electron micrograph of strain A23^T^, showing aerial mycelium that fragmented into rod-shaped elements after incubation on Organic medium 79 at 28 °C for 10 days

Cells of strain A23^T^ were aerobic, Gram-positive, non-motile. This strain showed a good growth on ISP 2-ISP 5 and moderate on both ISP 6 and ISP 7. The colours of substrate mycelia varied from light ivory to sulfur yellow while aerial hyphae usually were white or cream (Table 2). The branching mycelia (diameter 0.42 µm) fragmented into different lengths rod-shaped elements (Fig. 2), which is typically observed for *Amycolatopsis* (Franco and Labeda 2014).

**Table 2:** Growth and cultural characteristics of *Amycolatopsis* isolate A23^T^ after incubation for 14 days at 28 °C

Media	Growth	Aerial spore-mass	Substrate mycelia colour	Soluble pigment
Yeast extract-malt extract (ISP 2)	Good	White (9012)^a^	Beige (1001)	None
Oatmeal (ISP 3)	Very good	White (9012)	Honey yellow (1005)	None
Inorganic salts-starch (ISP 4)	Good	White (9012)	Light ivory (1015)	None
Glycerol-asparagine (ISP 5)	Good	Cream (9001)	Beige (1001)	Luminous bright orange (2007)
Peptone-yeast extract iron (ISP 6)	Moderate	White (9012)	Sun yellow (1037)	Sun yellow (1037)
Tyrosine (ISP 7)	Moderate	Cream (9001)	Sulfur yellow (1016)	Sulfur yellow (1016)
Organic medium 79	Good	White (9012)	Sun yellow (1037)	Sun yellow (1037)
Modified Bennett’s agar	Good	White (9012)	Suffon yellow (1017)	Suffon yellow (1017)

^a^Accordingly RAL colour standard

Strain A23^T^, as well as its closely related *A. australiensis* DSM 44671^T^, *A. balhimycina* DSM 44591^T^, *A. eburnea* NBRC 113658^T^, *A. kentuckyensis* NRRL B-24129^T^, *A. lexingtonensis* NRRL B-24131^T^, *A. mediterranei* NRRL B-3240^T^, *A. pretoriensis* DSM 44654^T^, *A. rifamycinica* DSM 46095^T^, *A. tolypomycina* DSM 44544^T^, *A. vancoresmycina* DSM 44592^T^, *A. vastitatis* NRRL B-65279^T^, produced acid from glucose and inositol. All the above mentioned strains were capable of peptonization of gelatin (Table 3). The majority of considered strains including strain A23^T^ were able to utilize arabinose, fructose, galactose, sucrose and decompose of urea. They grew at 30 °C and in the presence of NaCl 1% w/v, but none produced amylases. The optimum growth temperature and pH of strain A23^T^ were 28–30 °C and pH 7, accordingly, but it was able to grow between pH 6.0–9.0 and up to NaCl 5.0% (w/v).

**Table 3:** Differential characteristics of strain A23^T^ and its closely related species of *Amycolatopsis*

Property	1^a^	2^b^	3^b^	4^b^	5^b^	6^b^	7^b^	8^b^	9^b^	10^b^	11^b^	12^b^
Utilization activity												
Adonitol	−	−	n/d	+	+	+	−	−	+	n/d	n/d	n/d
Arabinose	+	+	+	+	+	+	+	+	+	+	+	n/d
Cellobiose	n/d	+	n/d	n/d	+	+	+	+	+	n/d	n/d	n/d
Fructose	+	+	+	n/d	+	+	+	+	n/d	+	+	−
Galactose	+	+	n/d	+	+	+	+	+	+	n/d	n/d	n/d
Glucose	+	+	+	+	+	+	+	+	+	+	+	+
Inositol	+	+	+	+	+	+	+	+	+	+	+	+
Lactose	+	+	n/d	+	+	+	+	+	−	−	−	−
Maltose	+	+	n/d	+	+	+	+	+	−	−	−	n/d
Mannitol	+	+	+	+	−	w	+	−	−	+	+	n/d
Raffinose	w	+	+	+	+	+	+	+	−	−	+	n/d
Rhamnose	+	−	+	+	+	+	+	+	−	+	+	n/d
Salicin	−	n/d	n/d	n/d	+	+	−	+	n/d	n/d	n/d	n/d
Sorbitol	+	−	−	+	+	−	−	w	−	n/d	n/d	n/d
Sucrose	+	+	+	n/d	+	+	+	+	n/d	+	+	n/d
Xylose	+	+	−	+	+	+	+	+	+	−	−	n/d
Growth in/at												
1% NaCl	+	+	n/d	n/d	+	+	+	+	+	n/d	n/d	+
5% NaCl	w	−	n/d	n/d	+	+	−	+	w	n/d	n/d	w
8% NaCl	−	−	n/d	n/d	−	n/d	−	−	−	n/d	n/d	−
10 °C	w	w	n/d	−	n/d	n/d	+	+	+	n/d	n/d	−
30 °C	+	+	n/d	+	+	+	+	+	+	n/d	n/d	+
40 °C	−	+	n/d	+	+	+	+	−	−	n/d	n/d	+
β-glucosidase	+	+	+	+	n/d	n/d	n/d	n/d	n/d	+	−	+
Arginine dihydrolase	+	n/d	−	n/d	n/d	n/d	n/d	n/d	n/d	+	+	−
Lysine decarboxylase	+	n/d	−	n/d	n/d	n/d	n/d	n/d	n/d	+	+	n/d
Ornithine decarboxylase	+	n/d	−	n/d	n/d	n/d	n/d	n/d	n/d	+	+	n/d
Citrate utilization	+	n/d	+	n/d	n/d	n/d	n/d	n/d	n/d	+	+	n/d
Decomposition of												
Casein	w	+	n/d	n/d	+	+	+	+	+	n/d	n/d	n/d
Cellulose	−	n/d	w	n/d	n/d	n/d	−	n/d	n/d	−	+	n/d
Gelatin	+	+	+	+	+	+	+	+	+	+	+	+
Starch	−	−	n/d	n/d	−	−	−	−	n/d	n/d	n/d	−
Urea	+	+	+	n/d	+	+	+	w	+	+	+	+

The strains were listed in the same order as in Table 1: + , positive; −, negative; w, weakly; n/d, not determined

^a^The data were obtained experimentally

^b^The sources were used for: 2, *A. australiensis* DSM 44671^T^ (Tan et al. 2006a); 3, *A. balhimycina* DSM 44591^T^, 10, *A. tolypomycina* DSM 44544^T^, 11, *A. vancoresmycina* DSM 44592^T^ (Wink et al. 2003); 4, *A. eburnea* NBRC 113658^T^ (Chaiya et al. 2019); 5, *A. kentuckyensis* NRRL B-24129^T^, 6, *A. lexingtonensis* NRRL B-24131^T^, 8, *A. pretoriensis* DSM 44654^T^ (Labeda et al. 2003); 7, *A. mediterranei* ATCC 13685^T^ (Lechevalier et al. 1986); 9, *A. rifamycinica* DSM 46095^T^ (Bala et al. 2004); 12, *A. vastitatis* NRRL B-65279^T^ (Idris et al. 2018)

The whole-cell hydrolysates of strain A23^T^ contained *meso*-2,6-diaminopimelic acid, arabinose, galactose, ribose and a trace of rhamnose as whole-cell sugars. However, strain DSM 44654^T^ had arabinose and galactose as the major cell sugars while ribose and rhamnose were present in traces. No mycolic acid was detected. Both strains showed similar isoprenoid quinone profile with MK-9(H4) as the predominant one (Table S3). The major fatty acids (> 10%) for strain A23^T^ were *iso*-C_16:0_, *iso*-C_15:0_, *anteiso*-C_17:0_ and C_16:0_ while the type strain of *A. pretoriensis* species had *iso*-C_16:0_ and *iso*-C_15:0_ (Table S4). The polar lipid pattern of A23^T^ included diphosphatidylglycerol, phosphatidylethanolamine (diagnostic lipid), aminophospholipid, a glycolipid, phospholipid and unidentified lipids (Fig. S6), as well as for strain DSM 44654^T^ (Fig. S7).

Online tool antiSMASH predicted 32 secondary metabolite gene clusters in the genome of strain A23^T^ (Table S5) and 30 ones in *A. pretoriensis* (Table S6). In total, from A23^T^ genome it was identified 11 PKS clusters, 10 NRPS and gene NRPS-like clusters, 2 RiPPs and 4 terpenes, while from DSM 44654^T^ there were 10, 10, 2 and 4 accordingly.

**Fig. 3:**
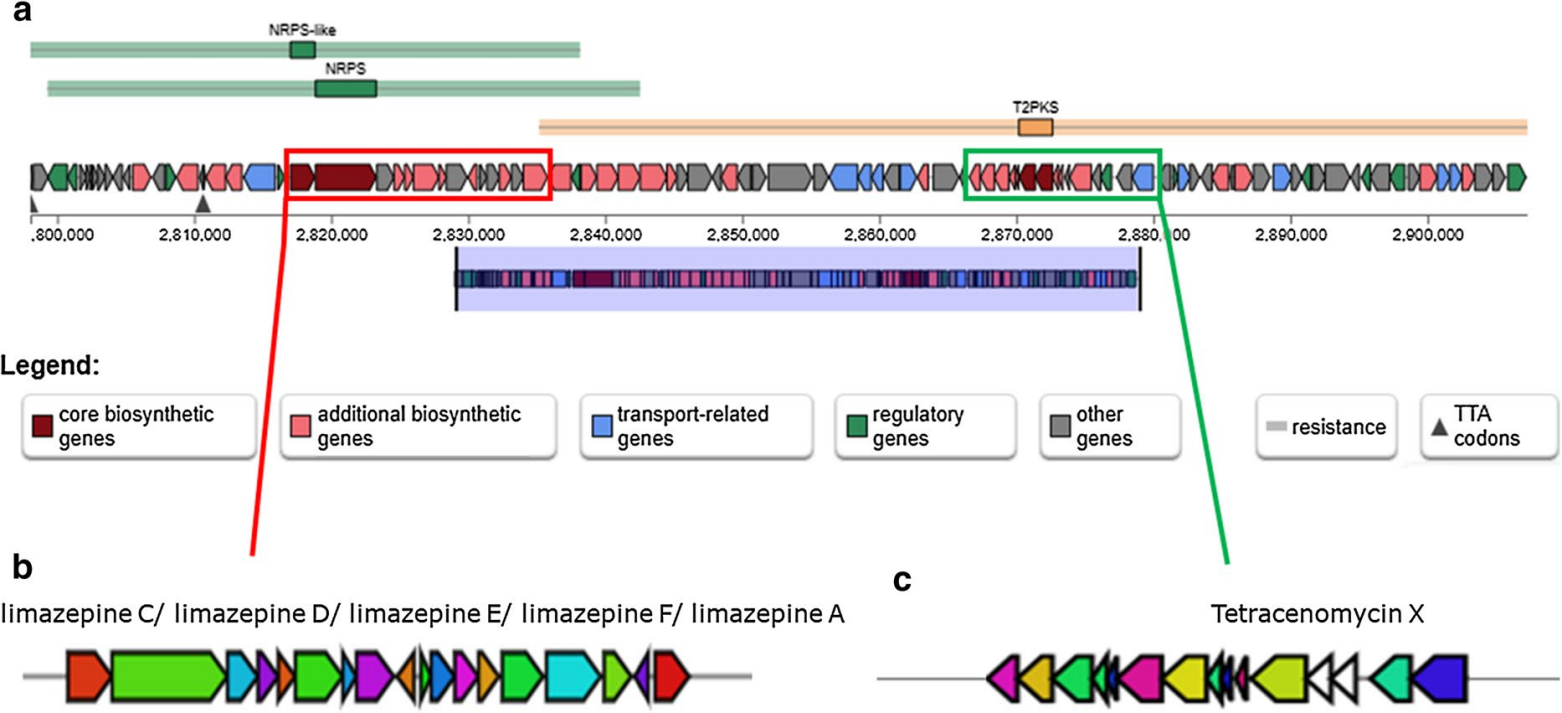
The BGCs in genome of *Amycolatopsis* A23^T^ **A** the structure of region 1.7 (location: 2,798,054–2,907,649 nt, total: 109,596 nt); **B** limazepines cluster of *Streptomyces* sp. ICBB 8177; **C** tetracenomycin X biosynthesis cluster

The genome mining of strain A23^T^ revealed that this strain has the potential to produce wide range of secondary metabolites including limazepines A, C-F and macrotermycins A-D (Table S5). The limazepines belong to the growing group of the pyrrolo[1,4]benzodiazepine antitumor antibiotics. Their inherent antitumor and antibacterial activities are due to their ability to regulate gene expression by recognizing and binding with DNA GC base pairs (Fotso et al. 2009). These limazepines’ clusters were identical to that in strain *Streptomyces* sp. ICBB 8177, and showed also 82% of genes similarity with the sequence of tomaymycin in strain *Streptomyces achromogenes* (Li et al. 2009) (Fig. 3). Macrotermycin biosynthetic gene clusters with 96% similarity to that of *Amycolatopsis* strain sp. M39 (Beemelmanns et al. 2017) were found in the genome of strains A23^T^ (Fig. S8). Macrotermycins A-D are 20-membered glycosylated polyketide macrolactams’s had antibacterial and also selective antifungal activity and were isolated from a termite-associated actinomycete, *Amycolatopsis* sp. M39 (Beemelmanns et al. 2017).

The DSM 44654^T^ genome contained the same clusters encoded limazepines and macrotermycins, supporting the view that phylogenetic similarity implies the presence of closely related biosynthetic pathways (Adamek et al. 2018).

Previously described tetracenomycin biosynthesis gene cluster and its paralogus Tcm2 cluster (Osterman et al. 2020) were detected in 1.7 and 2.5 regions of genome A23^T^ accordingly. Among all related strains *A. rifamycinica* DSM 46095^T^ and *A. balhimycina* DSM 44591^T^ have orthologous clusters in their genomes (Osterman et al. 2020). Despite the relatively closeness, no Tcm clusters were detected in the genome sequence of strain DSM 44654^T^ and other related strains.

The vast majority of *Amycolatopsis* from B-group were predicted as non human pathogen, the values of probability of being a human pathogen were below one (Table S7). Nevertheless, the protein families associated with pathogenicity were detected, so ABC-type transport system protein of *Saccharomonospora viridis* DSM 43017 was present in the genome of A23^T^ and all the strains examined (Table S7).

Based on phenotypic, phylogenetic and genomics analyses, strain A23^T^, producer of a valuable substance tetracenomycin X, is considered as a type strain of a novel species with the proposed name, *Amycolatopsis camponoti*.

### Description of *Amycolatopsis camponoti* sp. nov.

*Amycolatopsis camponoti* (cam.po.no′ti. N.L. gen. n. camponoti of *Camponotus*, referring to the insect *Camponotus vagus* Scopoli, from which the type strain was isolated).

Aerobic, Gram-strain-positive, non-motile and filamentous actinobacteria. The aerial mycelia fragments into rod-shaped fragments (0.42 µm in diameter). Well-developed substrate mycelium varies from light ivory to sulfur yellow, and the colour of aerial mycelium usually is white on ISP 2-ISP 4, ISP 6, MBA and Organic 79 media. When growing for three weeks in liquid Organic medium 79, it produces soluble pigments that ranges from faintly brown to red.

The optimum growth temperature and pH are 28–30 °C and pH 7, but it is unable to grow at 10 and 40 °C and out of range 6–9 pH same as above 5.0% salinity (w/v). It metabolizes arabinose, fructose, galactose, inositol, lactose, maltose, mannitol, rhamnose, sorbitol, sucrose, xylose and weakly raffinose, but unable to use adonitol, cellulose, starch and salicin. Strain A23^T^ demonstrates noticeable activity of β-glucosidase, arginine dihydrolase, lysine and ornithine decarboxylases.

The cell wall contains *meso*-2,6-diaminopimelic acid, arabinose, galactose, ribose and a trace of rhamnose as cell sugars. Major cellular fatty acids are *iso*-C_16:0_, *iso*-C_15:0_, *anteiso*-C_17:0_ and C_16:0_. The predominant menaquinone is MK-9(H4), while MK-9(H2) and MK-8(H4) are present as minor components.

The type strain is A23^T^ (= DSM 111725^T^ = VKM Ac-2882^T^), isolated from bodies of ants *Camponotus vagus* in Ryazan region, Russia. The genome size of the isolate A23^T^ is 10,560,374 bp with a DNA G + C content of 71.2%. The GenBank accession number for the 16S rRNA gene sequence and the genome assembly of strain A23^T^ are KY952635.2 and GCA_902497555, respectively.

## Supplementary Information

Below is the link to the electronic supplementary material.

Supplementary file1 (DOCX 6906 kb)

## Data Availability

The GenBank accession number for the 16S rRNA gene sequence and the genome assembly of strain A23^T^ are KY952635.2 and GCA_902497555, respectively. Detailed data on genome analysis A23^T^ are presented IMG/M DataBase (https://gold.jgi.doe.gov/).
