# Correction to: *Amycolatopsis camponoti* sp. nov., new tetracenomycin-producing actinomycete isolated from carpenter ant Camponotus vagus

**DOI:** 10.1007/s10482-022-01729-5

**Published:** 2022-04-10

**Authors:** Yuliya V. Zakalyukina, Ilya A. Osterman, Jacqueline Wolf, Meina Neumann-Schaal, Imen Nouioui, Mikhail V. Biryukov

**Affiliations:** 1 Scientific Center of Genetics and Life Sciences, Sirius University of Science and Technology, Sochi, Russia 354340; 2 Department of Soil Science, Lomonosov Moscow State University, Moscow, Russia 119991; 3 Skolkovo Institute of Science and Technology, Skolkovo, Moscow Region Russia 143025; 4 Department of Chemistry and A.N. Belozersky Institute of Physico-Chemical Biology, Lomonosov Moscow State University, Moscow, Russia 119991; 5 Leibniz Institute DSMZ–German Collection of Microorganisms and Cell Cultures, 38124 Braunschweig, Germany; 6 Department of Biology, Lomonosov Moscow State University, Moscow, Russia 119991

## Correction to: Antonie van Leeuwenhoek (2022) 115:533–544 https://doi.org/10.1007/s10482-022-01716-w

In the original publication of the article, the number of the strain in VKM was incorrectly published as “VKM 2882^T^”. It should be “VKM Ac-2882^T^”.

And the missing funding information “This research was funded by Sirius University (project BTH-RND-2127) for YVZ and by Ministry of Science and Higher Education of the Russian Federation (Agreement № 075-15-2021-1085) for IAO, MVB” are included.

The original article has been corrected.

