# Correction: *Amycolatopsis camponoti* sp. nov., new tetracenomycin‑producing actinomycete isolated from carpenter ant *Camponotus vagus*

**DOI:** 10.1007/s10482-023-01815-2

**Published:** 2023-03-22

**Authors:** Yuliya V. Zakalyukina, Ilya A. Osterman, Jacqueline Wolf, Meina Neumann‑Schaal, Imen Nouioui, Mikhail V. Biryukov

**Affiliations:** 1 Scientifc Center of Genetics and Life Sciences, Sirius University of Science and Technology, Sochi, Russia 354340; 2 Department of Soil Science, Lomonosov Moscow State University, Moscow, Russia 119991; 3 Skolkovo Institute of Science and Technology, Skolkovo, Moscow Region Russia 143025; 4 Department of Chemistry and A.N. Belozersky Institute of Physico‑Chemical Biology, Lomonosov Moscow State University, Moscow, Russia 119991; 5 Leibniz Institute DSMZ–German Collection of Microorganisms and Cell Cultures, 38124 Brunswick, Germany; 6 Department of Biology, Lomonosov Moscow State University, Moscow, Russia 119991

**Correction to: Antonie van Leeuwenhoek (2022) 115:533–544** **https://doi.org/10.1007/s10482-022-01716-w**

In the original publication of the article, the VKM culture collection number of the strain was incorrectly published as “VKM 2882^T^”. This error was rectified in the original article as “VKM Ac-2882^T^” and a correction article was published separately indicating the changes made in the original article.

However, to comply with the rules of the International Code of Nomenclature of Prokaryotes (ICNP), the author would like to reverse the correction updated in the original article and issue a corrigendum containing the full corrected protologue.

The original version of the article is restored and a new correction article is published as below.

In abstract of the paper, the sentence “Based on the phenotypic, genomic and phylogenetic data, isolate A23^T^ represents a novel species of the genus *Amycolatopsis*, for which the name *Amycolatopsis camponoti* sp. nov. is proposed, and the type strain is A23^T^ (= DSM 111725^T^ = VKM 2882^T^).” should read “Based on the phenotypic, genomic and phylogenetic data, isolate A23^T^ represents a novel species of the genus *Amycolatopsis*, for which the name *Amycolatopsis camponoti* sp. nov. is proposed, and the type strain is A23^T^ (= DSM 111725^T^ = VKM Ac-2882^T^).”

The correct protologue for this novel taxon should read:

**Description of Amycolatopsis camponoti sp. nov.**

*Amycolatopsis camponoti* (cam.po.no′ti. N.L. gen. n. *camponoti* of *Camponotus*, referring to the insect *Camponotus vagus* Scopoli, from which the type strain was isolated).

Aerobic, Gram-strain-positive, non-motile and filamentous actinobacteria. The aerial mycelia fragments into rod-shaped fragments (0.42 µm in diameter). Well-developed substrate mycelium varies from light ivory to sulfur yellow, and the colour of aerial mycelium usually is white on ISP 2-ISP 4, ISP 6, MBA and Organic 79 media. When growing for three weeks in liquid Organic medium 79, it produces soluble pigments that ranges from faintly brown to red.

The optimum growth temperature and pH are 28–30 °C and pH 7, but it is unable to grow at 10 and 40 °C and out of range 6–9 pH same as above 5.0% salinity (w/v). It metabolizes arabinose, fructose, galactose, inositol, lactose, maltose, mannitol, rhamnose, sorbitol, sucrose, xylose and weakly raffinose, but unable to use adonitol, cellulose, starch and salicin. Strain A23^T^ demonstrates noticeable activity of β-glucosidase, arginine dihydrolase, lysine and ornithine decarboxylases.

The cell wall contains *meso*-2,6-diaminopimelic acid, arabinose, galactose, ribose and a trace of rhamnose as cell sugars. Major cellular fatty acids are *iso*-C_16:0_, *iso*-C_15:0_, *anteiso*-C_17:0_ and C_16:0_. The predominant menaquinone is MK-9(H4), while MK-9(H2) and MK-8(H4) are present as minor components.

The type strain is A23^T^ (= DSM 111725^T^ = VKM Ac-2882^T^), isolated from bodies of ants *Camponotus vagus* in Ryazan region, Russia. The genome size of the isolate A23^T^ is 10,560,374 bp with a DNA G + C content of 71.2%. The GenBank accession number for the 16S rRNA gene sequence and the genome assembly of strain A23^T^ are KY952635.2 and GCA_902497555, respectively.

