# Supplementary material for: Amycolatopsis camponoti sp. nov., new tetracenomycin-producing actinomycete isolated from carpenter ant Camponotus vagus
Source: Antonie Van Leeuwenhoek. 2022 Feb 26;115(4):533–44. doi: 10.1007/s10482-022-01716-w (PMC8930869; doi:10.1007/s10482-022-01716-w)
Supplement: Supplementary file 1 — Supplementary file1 (DOCX 6906 kb) [file 10482_2022_1716_MOESM1_ESM.docx]

*Amycolatopsis camponoti* sp.nov., new tetracenomycin-producing actinomycete isolated from carpenter ant *Camponotus vagus*

Supplementary information

**Yuliya V. Zakalyukina^1,2^*, Iliya A. Osterman ^1,3,4^, Jacqueline Wolf^5^, Meina Neumann-Schaal^5^, Imen Nouioui^5^ and Mikhail V. Biryukov^1,3,6^**

^1^ Scientific Center of Genetics and Life Sciences, Sirius University of Science and Technology, Sochi, 354340, Russia

^2^ Department of Soil Science, Lomonosov Moscow State University, Moscow, 119991, Russia; juline@soil.msu.ru

^3^ Skolkovo Institute of Science and Technology, Skolkovo, Moscow Region, 143025, Russia; osterman@yandex.ru

^4^ Department of Chemistry and A.N. Belozersky Institute of Physico-Chemical Biology, Lomonosov Moscow State University, Moscow, 119991, Russia

^5^ Leibniz Institute DSMZ–German Collection of Microorganisms and Cell Cultures, 38124 Braunschweig, Germany

^6^ Department of Biology, Lomonosov Moscow State University, Moscow, 119991, Russia; metrim@gmail.com

***** Correspondence: juline@soil.msu.ru (Y.V.Z.); Tel.: +79175548004 (Y.V.Z)

Contents

[Fig. S1 Neighbour-joining phylogenetic tree of strain A23^T^ and related *Amycolatopsis* species, 3](#_Toc94134373)

[Fig. S2 Maximum-likelihood phylogenetic tree of strain A23^T^ 4](#_Toc94134374)

[Fig. S3 Maximum-parsimony phylogenetic tree 5](#_Toc94134375)

[Table S1. Genome relatedness of A23^T^ and *Amycolatopsis* type-strains 6](#_Toc94134376)

[Table S2. The estimation of sequence similarity between full-length 16S rRNA gene sequences and sequences obtained through the Sanger method for strain A23^T^ and closely related *Amycolatopsis species* 7](#_Toc94134377)

[Table S3. The composition of respiratory quinones (%) of strain A23^T^ and closely related *A. pretoriensis* DSM 44654^T^ 8](#_Toc94134378)

[Table S4. Fatty acid content of A23^T^ and closely related *A. pretoriensis* DMS 44654^T^ 9](#_Toc94134379)

[Fig. S4 Spectrum of some carbohydrate utilization of A23^T^ 10](#_Toc94134380)

[Fig. S5 TLC plates of whole cell sugars of A23^T^ and its closely related neighbour *A. pretoriensis* DMS 44654^T^. 11](#_Toc94134381)

[Fig. S6. The polar phospholipids of strain A23T: 12](#_Toc94134382)

[Fig. S7. The polar phospholipids of strain *A. pretoriensis* DMS 44654^T^.: 13](#_Toc94134383)

[Table S5. Secondary metabolite gene clusters in *Amycolatopsis* A23^T^ 14](#_Toc94134384)

[Table S6. Secondary metabolite gene clusters in *Amycolatopsis pretoriensis* DSM 44654^T^ 15](#_Toc94134385)

[Fig. S8 The genetic map of the macrotermycin (Mte) biosynthetic gene cluster: 16](#_Toc94134386)

[Table S7. Probability of being a human pathogen: family and function of pathogenic proteins in *Amycolatopsis* genomes 17](#_Toc94134387)

# Fig. S1 Neighbour-joining phylogenetic tree of strain A23^T^ and related *Amycolatopsis* species,

calculated from 16S rRNA full-length sequences. The evolutionary distances were computed using the Tamura-Nei method. Bootstrap values for n=1,000 are given at the branching points in percentages for values higher than 60. Bar, 0,01 nucleotide substitutions per site. There were a total of 1414 positions in the final dataset. *Streptomyces alboniger* DSM 40043^T^ was used as the outgroup.


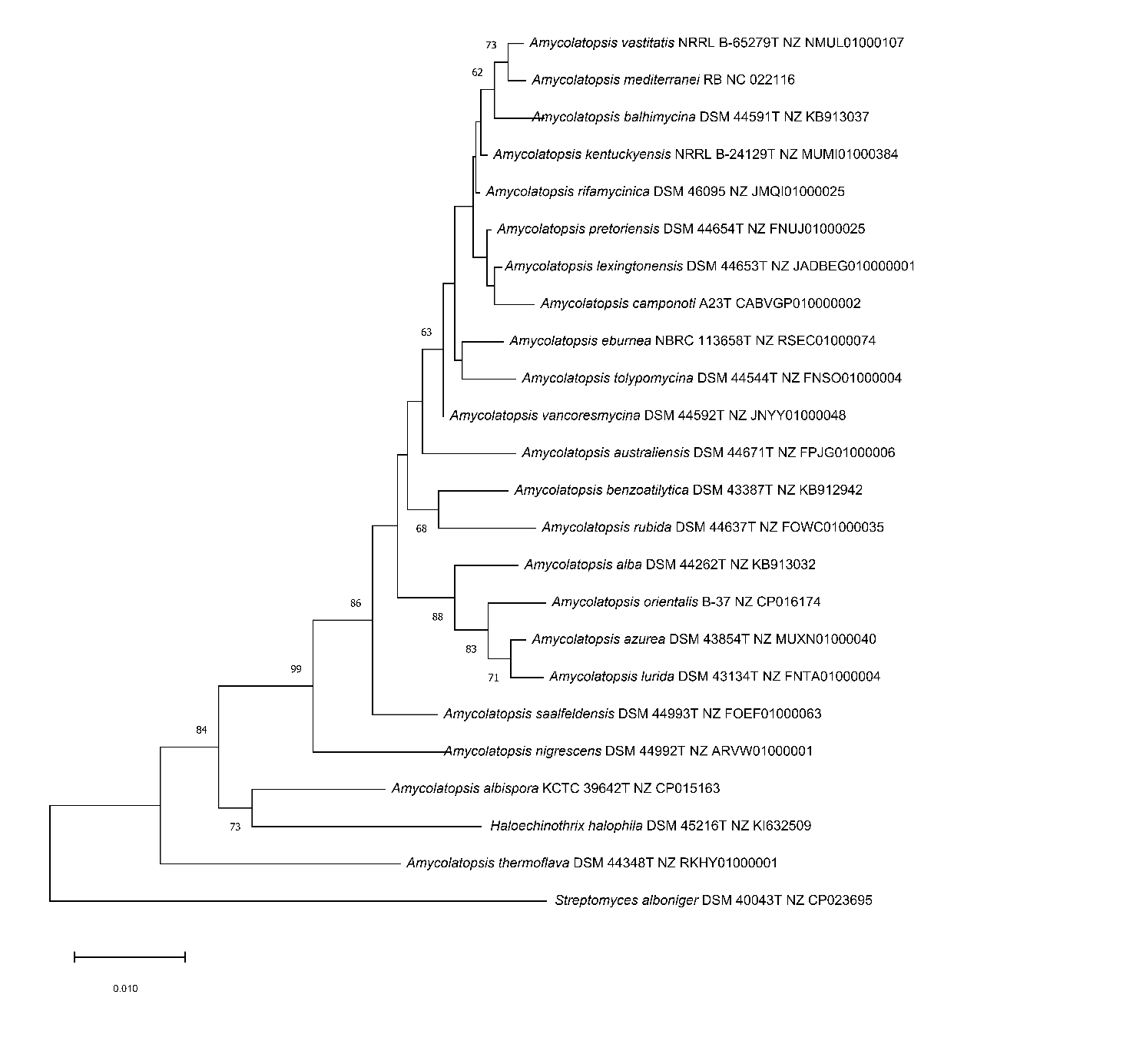


# Fig. S2 Maximum-likelihood phylogenetic tree of strain A23^T^

and comparison with related *Amycolatopsis* species, based on almost complete 16S rRNA gene sequences. Bootstrap percentages based on 1000 resamplings are listed at the nodes (only value >60% are shown). The percentage of trees in which the associated taxa clustered together is shown next to the branches. Initial tree(s) for the heuristic search were obtained automatically by applying Neighbor-Join and BioNJ algorithms to a matrix of pairwise distances estimated using the Tamura-Nei model, and then selecting the topology with superior log likelihood value. Bar, 0.01 substitutions per nucleotide position. *Streptomyces alboniger* DSM 40043^T^ was used as the outgroup.


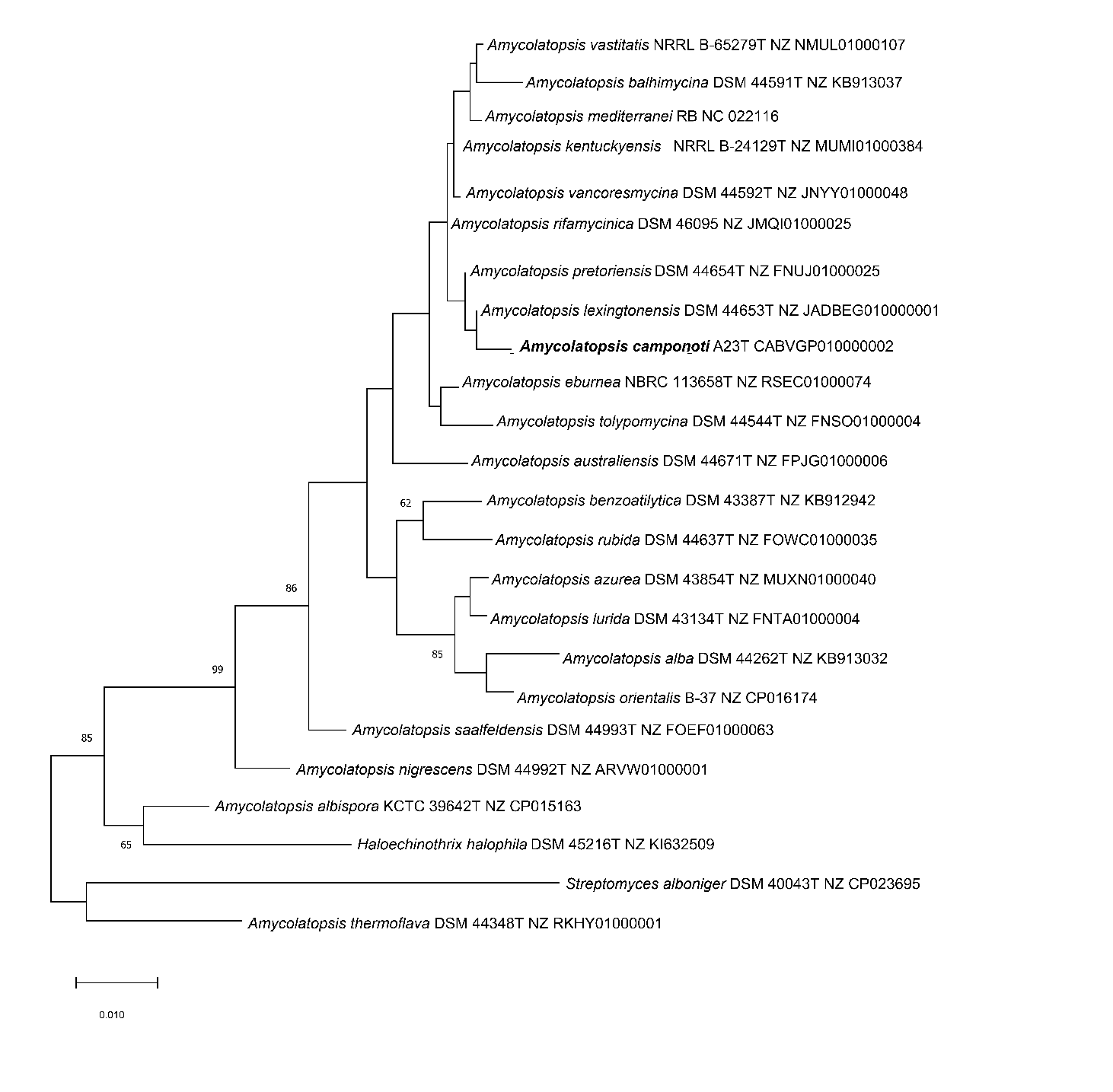


# Fig. S3 Maximum-parsimony phylogenetic tree

based on almost complete 16S rRNA gene sequences showing the position of strain A23^T^ among the related type species of the genus *Amycolatopsis*. Bootstrap percentages based on 1000 resamplings are listed at the nodes (only value >60% are shown). There were a total of 1414 positions in the final dataset.


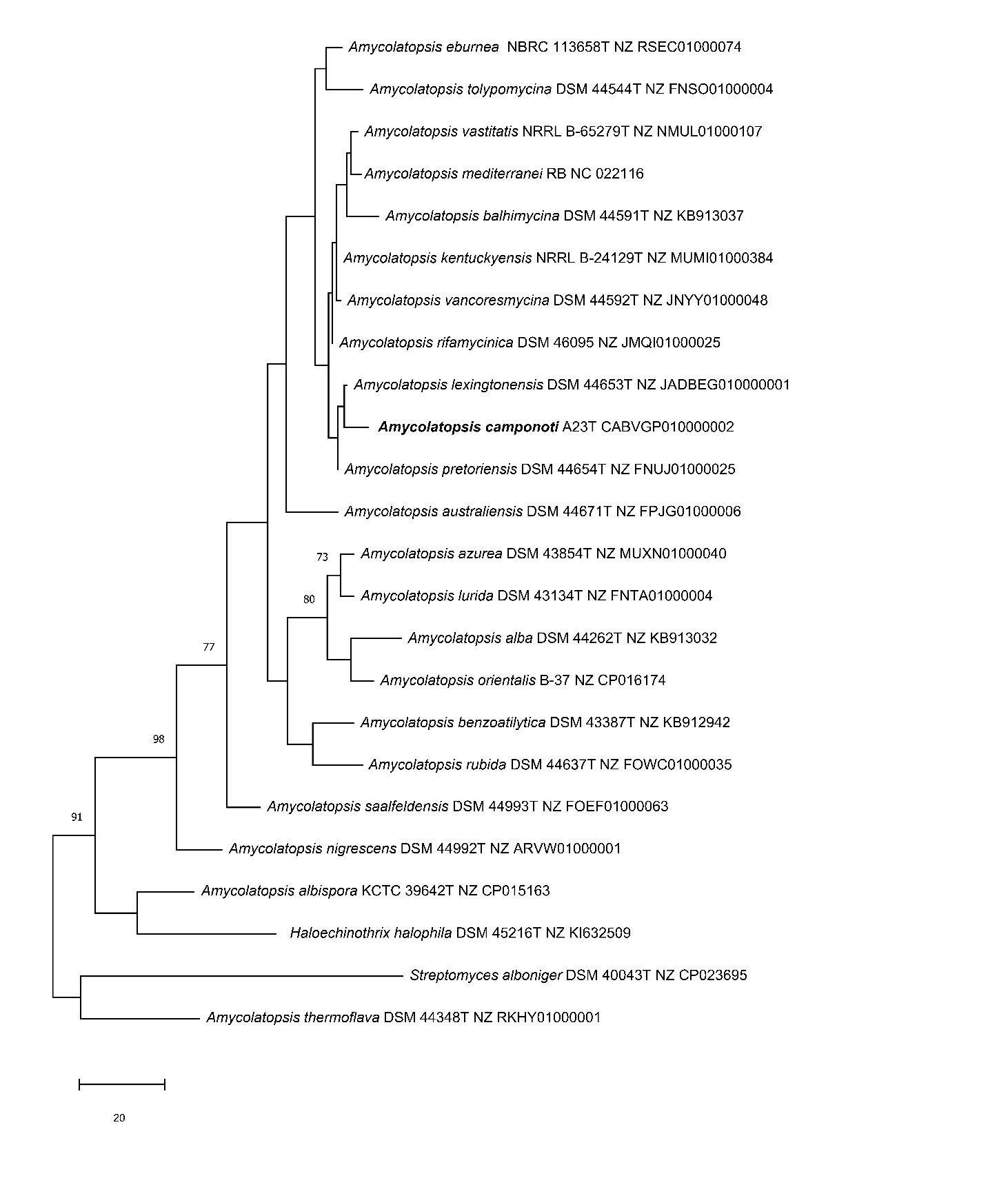


# Table S1. Genome relatedness of A23^T^ and *Amycolatopsis* type-strains

| **Subject strain** | **ANIb, %** | **dDDH (in %)** | **G+C content difference (in %)** |
| --- | --- | --- | --- |
| *A. australiensis* DSM 44671^T^ | 86.45 | 35.6 | 0.71 |
| *A. balhimycina* DSM 44591^T^ | 85.58 | 34.0 | 0.40 |
| *A. eburnea* NBRC 113658^T^ | 85.80 | 34.7 | 0.68 |
| *A. kentuckyensis* NRRL B-24129^T^ | 85.86 | 34.2 | 0.57 |
| *A. lexingtonensis* NRRL B-24131^T^ | 86.83 | 35.4 | 0.37 |
| *A. mediterranei* NRRL B-3240 ^T^ | 85.82 | 34.1 | 0.12 |
| *A. pretoriensis* DSM 44654^T^ | 88.57 | 39.5 | 0.00 |
| *A. rifamycinica* DSM 46095^T^ | 85.50 | 33.6 | 0.68 |
| *A. tolypomycina* DSM 44544^T^ | 85.43 | 33.1 | 0.51 |
| *A. vancoresmycina* DSM 44592^T^ | 85.63 | 33.4 | 0.81 |
| *A. vastitatis* NRRL B-65279^T^ | 85.52 | 34.4 | 0.32 |
| *Amycolatopsis* sp. A23^T^ | 100 | 100 | 0.0 |

# Table S2. The estimation of sequence similarity between full-length 16S rRNA gene sequences and sequences obtained through the Sanger method for strain A23^T^ and closely related *Amycolatopsis species*

| Strain | Whole genome number in GenBank | 16SrRNA | Identities | Gaps |
| --- | --- | --- | --- | --- |
| *Amycolatopsis eburnea* NBRC 113658^T^ | NZ_RSEC01000074 | MH598363.1 | 1400/1403(99%) | 0/1403(0%) |
| *Amycolatopsis alba* DSM 44262^T^ | NZ_KB913032 | NR_024888.1 | 1473/1478(99%) | 1/1478(0%) |
| KCTC 39642^T^ | NZ_CP015163 | NR_152021.1 | 1476/1481(99%) | 0/1481(0%) |
| *Amycolatopsis australiensis* DSM 44671^T^ | NZ_FPJG01000006 | NR_042746.1 | 1459/1462(99%) | 1/1462(0%) |
| *Amycolatopsis azurea* DSM 43854^T^ | NZ_MUXN01000040 | NR_118889.1 | 1252/1303(96%) | 3/1303(0%) |
| *Amycolatopsis balhimycina* DSM 44591^T^ | NZ_KB913037 | NR_025564.1 | 1448/1450(99%) | 1/1450(0%) |
| *Amycolatopsis benzoatilytica* DSM 43387^T^ | NZ_KB912942 | NR_115344.1 | 1375/1416(97%) | 24/1416(1%) |
| *Amycolatopsis kentuckyensis* NRRL B-24129^T^ | NZ_MUMI01000384 | NR_029068.1 | 1502/1506(99%) | 1/1506(0%) |
| *Amycolatopsis lexingtonensis* DSM 44653^T^ | NZ_JADBEG010000001 | NR_042765.1 | 1440/1444(99%) | 0/1444(0%) |
| *Amycolatopsis lurida* DSM 43134^T^ | NZ_FNTA01000004 | NR_042040.1 | 1489/1489(100%) | 0/1489(0%) |
| *Amycolatopsis mediterranei RB* | NC_022116 | NR_115160.1 | 1482/1482(100%) | 0/1482(0%) |
| *Amycolatopsis nigrescens* DSM 44992T | NZ_ARVW01000001 | NR_043880.1 | 1408/1409(99%) | 1/1409(0%) |
| *Amycolatopsis orientalis* B-37 | NZ_CP016174 | NR_042104.1 | 1443/1458(99%) | 0/1458(0%) |
| *Amycolatopsis pretoriensis* DSM 44654^T^ | NZ_FNUJ01000025 | NR_025693.1 | 1398/1398(100%) | 0/1398(0%) |
| *Amycolatopsis rifamycinica* DSM 46095 | NZ_JMQI01000025 | NR_029049.1 | 1376/1377(99%) | 0/1377(0%) |
| *Amycolatopsis rubida* DSM 44637^T^ | NZ_FOWC01000035 | NR_025072.1 | 1397/1407(99%) | 5/1407(0%) |
| *Amycolatopsis saalfeldensis* DSM 44993^T^ | NZ_FOEF01000063 | NR_043964.1 | 1420/1424(99%) | 3/1424(0%) |
| *Amycolatopsis thermoflava* DSM 44348^T^ | NZ_RKHY01000001 | NR_024890.1 | 1480/1496(99%) | 2/1496(0%) |
| *Amycolatopsis tolypomycina* DSM 44544^T^ | NZ_FNSO01000004 | NR_114882.1 | 1404/1408(99%) | 0/1408(0%) |
| *Amycolatopsis vancoresmycina* DSM 44592^T^ | NZ_JNYY01000048 | NR_025565.1 | 1296/1298(99%) | 1/1298(0%) |
| *Amycolatopsis vastitatis* NRRL B-65279^T^ | NZ_NMUL01000107 | NR_164904.1 | 1416/1427(99%) | 3/1427(0%) |

# Table S3. The composition of respiratory quinones (%) of strain A23^T^ and closely related *A. pretoriensis* DSM 44654^T^

| Strains | MK-9(H2) | MK-9(H4) | MK-9(H6) | MK-9(H8) | MK-8(H4) | MK-10(H4) |
| --- | --- | --- | --- | --- | --- | --- |
| A23^T^ | 5.2 | 83.3 | 3.5 | 1.6 | 6.4 | nd^1^ |
| DSM 44654^T^ | 9.4 | 78.4 | 4.6 | 0.1 | 6.1 | 1.4 |

^1^ nd – not detected

# Table S4. Fatty acid content (%)* of A23^T^ and closely related *A. pretoriensis* DMS 44654^T^

| Fatty acids | A23^T^ | DMS 44654^T^ |
| --- | --- | --- |
| *iso*-C_13:0_ | 0.16 | — |
| *anteiso*-C_13:0_ | 0.13 | — |
| iso-C_14:0_ | 1.93 | 0.91 |
| C_14:0_ | 0.25 | — |
| iso-C_15:0_ | 18.41 | 13.11 |
| anteiso-C_15:0_ | 6.08 | 1.53 |
| C_15:1_ *cis* 9 | 0.33 | — |
| С_15:0_ | 2.83 | 1.35 |
| iso-C_16:1_ *cis* 9 | 0.29 | 0.21 |
| iso-C_16:0_ | 21.87 | 35.32 |
| anteiso-C_16:0_ | 0.3 | 0.18 |
| C_16:1_ *cis* 9 | 1.86 | — |
| ** iso-C_15:0_ 2OH | 1.11 | 3.66 |
| anteiso-C_15:0_ 2OH | 0.42 | 0.64 |
| C_16:0_ | 10.38 | 3.34 |
| ** iso-C_16:1_  *cis* 9 9-Methyl | 1.21 | 0.87 |
| C_16:0_ 10 Methyl | 1.49 | 0.56 |
| anteiso-C_17:1_ C | 0.36 | — |
| iso-C_17:0_ | 3.19 | 5.61 |
| anteiso-C_17:0_ | 11.48 | 6.07 |
| C_17:1_ *cis* 9 | 2.55 | 0.8 |
| iso-C_16:0_ 2OH | 0.88 | 6.50 |
| C_17:0_ | 7.59 | 6.72 |
| ** iso-C_17:1_ *cis* 9 9-Methyl | 1.75 | 7.34 |
| C_17:0_ 10 Methyl | 0.91 | 1.95 |
| iso-C_18:0_ | 0.13 | 1.4 |
| C_18:1_ *cis* 9 | 0.17 | — |
| C_18:0_ | 1.12 | 1.74 |

* minor components (less 0.1%) are not shown

** identified as

# Fig. S4 Spectrum of some carbohydrate utilization of A23^T^

**
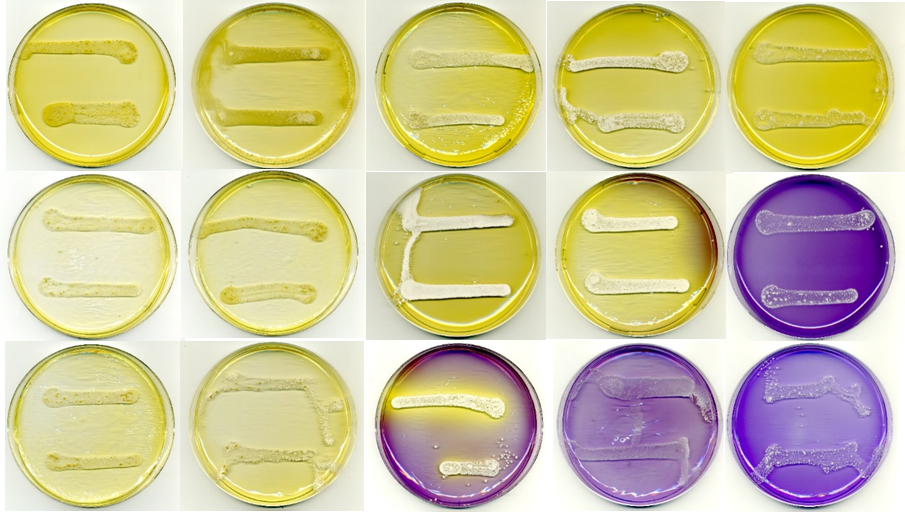
**

Top row, left to right: glucose, fructose, arabinose, maltose, rhamnose;

middle row, left to right: galactose, lactose, sucrose, inositol, cellulose;

bottom row: mannitol, xylose, raffinose, sorbitol, mineral base without sugar.

# Fig. S5 TLC plates of whole cell sugars of A23^T^ and its closely related neighbour *A. pretoriensis* DMS 44654^T^.

RHA, rhamnose, GAL, galactose, RIB, ribose, ARA, arabinose, Sd, standard

# Fig. S6 The polar phospholipids of strain A23^T^:

a, using naphthol acid reagent; b, using dodeca-molybdophosphoric acid; c, using ninhydrin reagent; d, using ninhydrin (O)/molybdenum blue (\\\).

Abbreviations: DPG, diphosphatidylglycerol; PE, phosphatidylethanolamine; APL, aminophospholipid; GL, glycolipid; PL, phospholipid; L, lipid

| 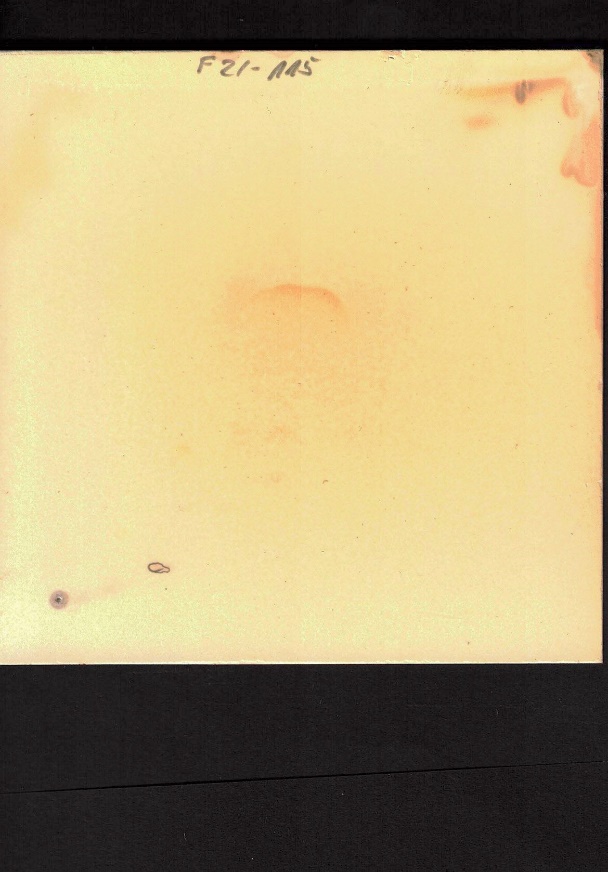 | 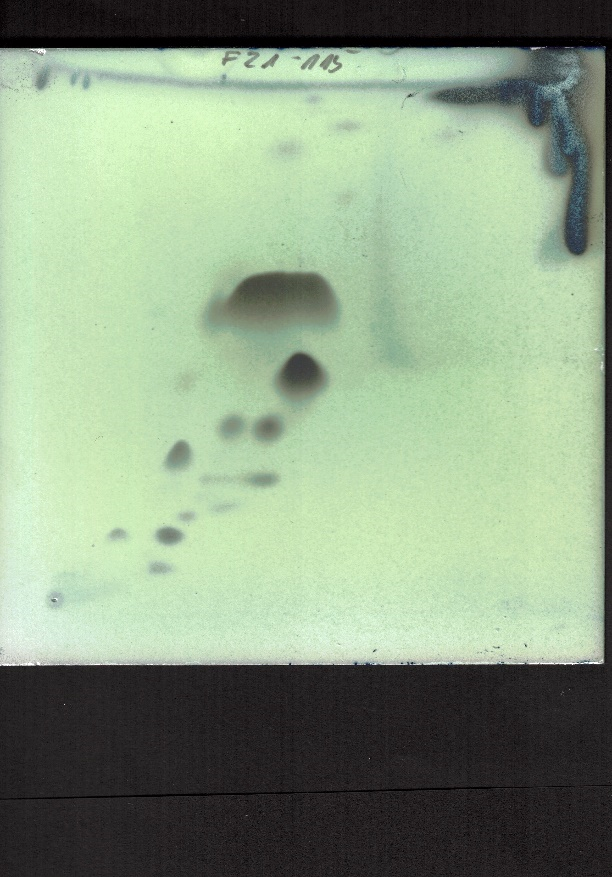 PE  DPG  APL  GL  APL  PL  L  L  L  PL  PL  PL  PL  APL  L |
| --- | --- |
| a | b |
| 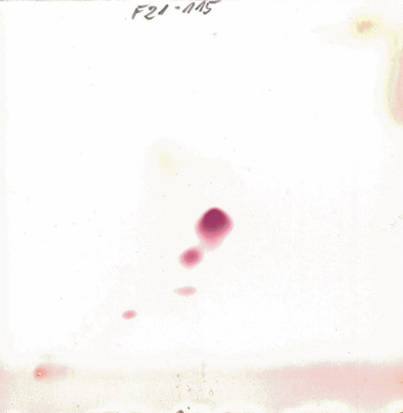 | 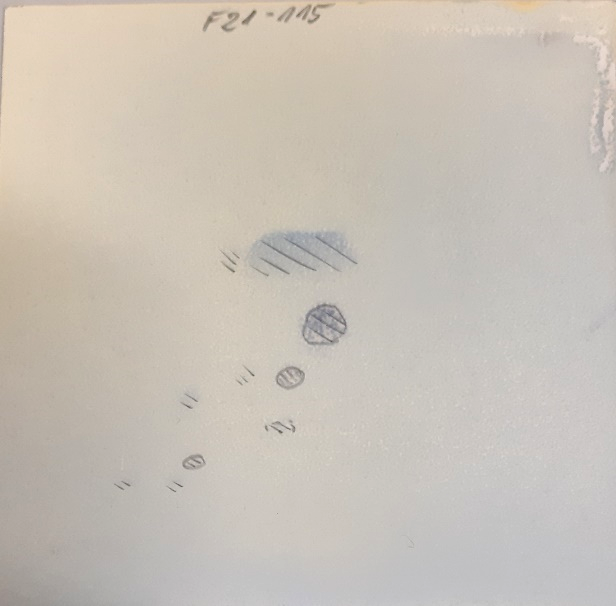 |
| c | d |

# Fig. S7 The polar phospholipids of strain *A. pretoriensis* DMS 44654^T^.:

a, using naphthol acid reagent; b, using dodeca-molybdophosphoric acid; c, using ninhydrin reagent; d, using ninhydrin (O)/molybdenum blue (\\\).

Abbreviations: DPG, diphosphatidylglycerol; PE, phosphatidylethanolamine; APL, aminophospholipid; GL, glycolipid; PL, phospholipid; L, lipid

| 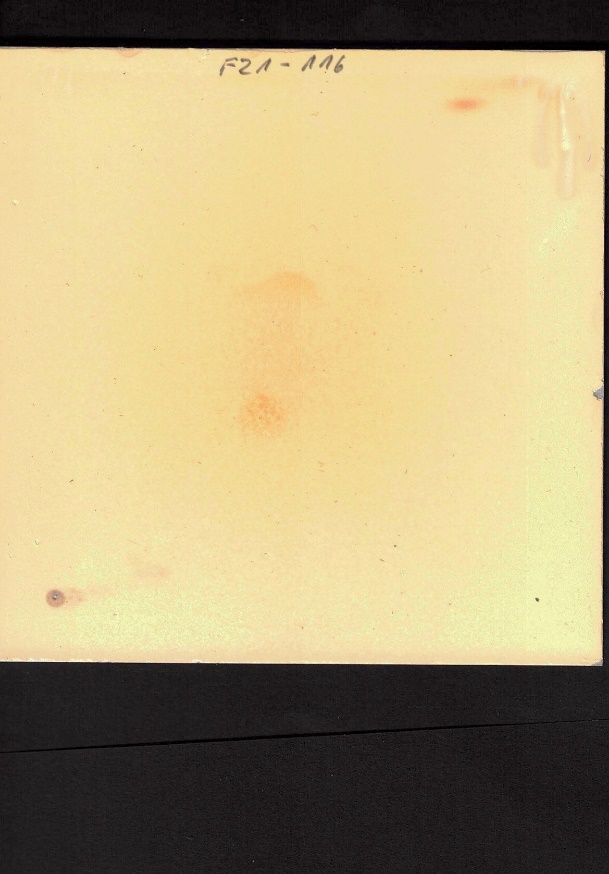 | 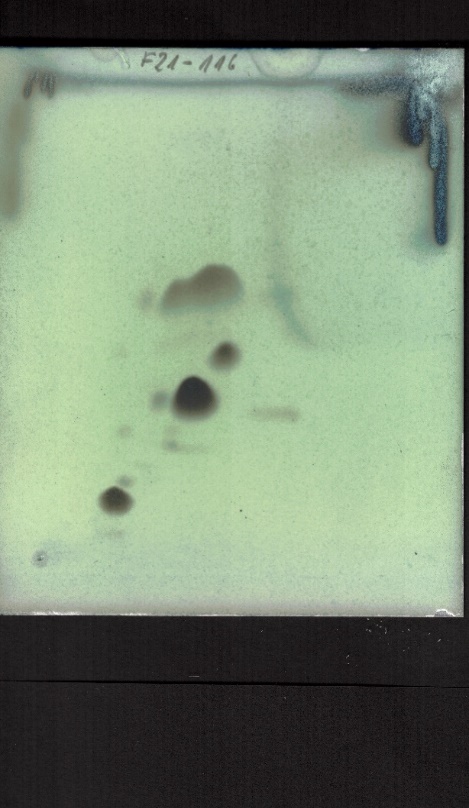 PE  APL  DPG  L  AL  PL  AL  PL  PL  APL |
| --- | --- |
| a | b |
| 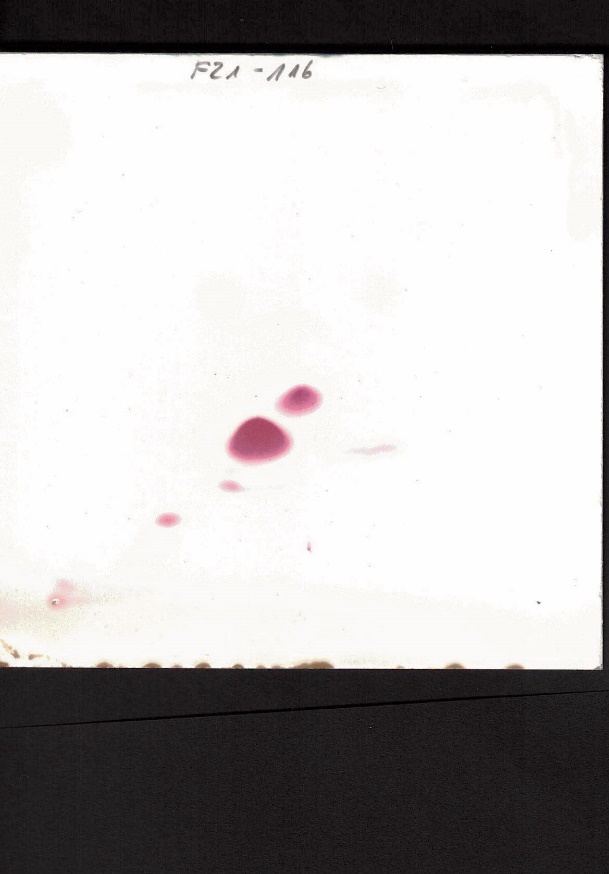 | 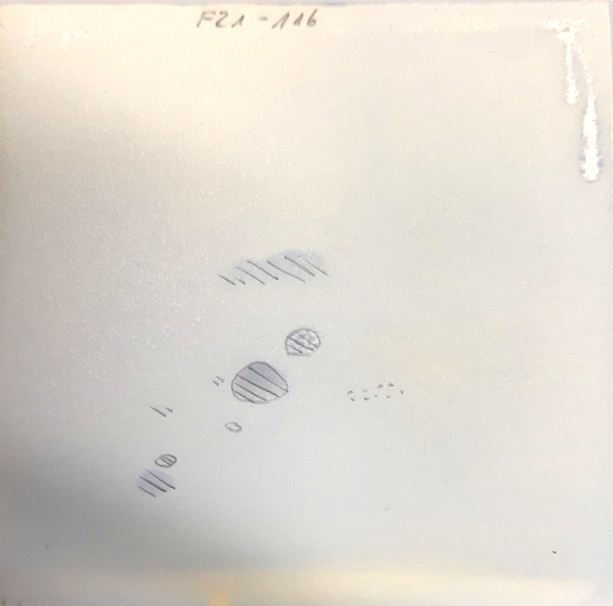 |
| c | d |

# Table S5. Secondary metabolite gene clusters in *Amycolatopsis* A23^T^

| Region | Type | From (bp) | To (bp) | Products of most similar cluster | Similarity,% |
| --- | --- | --- | --- | --- | --- |
| 1.1 | ectioin | 38,513 | 48,905 | Ectoine | 100 |
| 1.2 | NRP+Polyketide | 804,440 | 826,441 | Linkacidin C | 20 |
| 1.3 | Polyketide | 1,012,995 | 1,083,802 | Rifamorpholines A-E | 9 |
| 1.4 | Terpene | 1,750,993 | 1,770,613 | Isorenieratene | 42 |
| 1.5 | RiPP-like | 2,135,622 | 2,145,140 |  |  |
| 1.6 | Terpene | 2,562,204 | 2,582,452 | Isorenieratene | 71 |
| 1.7 | NRP | 2,798,054 | 2,907,649 | Limazepines A, C-F | 100 |
| 1.8 | Terpene | 2,973,065 | 2,994,180 | 2-methylisoborneol | 100 |
| 1.9 | RiPP-like | 3,080,354 | 3,092,141 |  |  |
| 1.10 | Polyketide | 3,147,520 | 3,193,172 | Niphimycins C-E | 9 |
| 1.11 | Polyketide | 3,323,398 | 3,367,527 | Saquayamycin A | 7 |
| 1.12 | Polyketide | 3,958,821 | 4,001,430 |  |  |
| 1.13 | Polyketide | 4,152,698 | 4,203,838 |  |  |
| 1.14 | NRP | 4,302,110 | 4,374,525 | Dechlorocuracomycin | 12 |
| 2.1 | Terpene | 137,529 | 159,595 | Geosmin | 100 |
| 2.2 | Butyrolactone | 238,046 | 341,027 | Macrotermycins | 96 |
| 2.3 | Terpene | 1,179,816 | 1,257,455 | Amycolamycins A,B | 45 |
| 2.4 | Ectoine | 1,263,567 | 1,273,938 | Kosinostatin | 6 |
| 2.5 | Polyketide | 1,523,632 | 1,598,321 | FD-594 | 26 |
| 2.6 | Polyketide | 1,615,273 | 1,781,609 | Aculeximycin | 61 |
| 2.7 | Saccharide | 1,784,450 | 1,805,529 | Fortimicin | 4 |
| 2.8 | NAPAA | 2,199,665 | 2,233,600 |  |  |
| 2.9 | NRP+Polyketide | 2,261,757 | 2,283,423 | FR-900520 | 9 |
| 2.10 | NRP | 2,889,117 | 2,915,073 |  |  |
| 2.11 | NRP | 3,000,765 | 3,052,856 | JBIR-126 | 7 |
| 2.12 | NRP | 3,076,766 | 3,127,493 | rimosamide | 21 |
| 2.13 | NRP | 3,146,009 | 3,210,324 | atratumycin | 21 |
| 2.14 | Lanthipeptide II | 3,230,071 | 3,294,048 | Friulimicins A-D | 21 |
| 2.15 | Redox-cofactor | 3,334,745 | 3,360,384 |  |  |
| 3.1 | Lanthipeptide I | 98,996 | 124,294 |  |  |
| 3.2 | Lanthipeptide III | 1,309,125 | 1,331,689 | Ery3-9 | 100 |
| 4.1 | NRP | 353,119 | 411,166 | scabechelin | 80 |

NRP - non-ribosomal peptide, RiPP-like - other unspecified ribosomally synthesised and post-translationally modified peptide product (RiPP) cluster, NAPAA - non-alpha poly-amino acids like e-Polylysin

# Table S6. Secondary metabolite gene clusters in *Amycolatopsis pretoriensis* DSM 44654^T^

| Region | Type | From (bp) | To (bp) | Most similar known cluster | Similarity |
| --- | --- | --- | --- | --- | --- |
| 1.1 | terpene | 1 | 11,224 | 2-methylisoborneol | 50 |
| 1.2 | betalactone | 14,378 | 39,530 |  |  |
| 1.3 | T1PKS | 315,426 | 360,291 | saquayamycin A | 7 |
| 1.4 | T1PKS | 872,299 | 915,225 | primycin | 5 |
| 1.5 | hglE-KS, T1PKS | 1,090,289 | 1,141,330 | meilingmycin |  |
| 2.1 | LAP, thioamitides | 191,773 | 224,955 |  |  |
| 2.2 | redox-cofactor | 380,526 | 406,476 |  |  |
| 2.3 | NRPS, lanthipeptide class-II | 493,669 | 556,944 | friulimicins A-D | 21 |
| 2.4 | NRPS-like, oligosaccharide, NRPS, T2PKS, T1PKS, RiPP-like | 681,768 | 833,721 | arixanthomycins A-C | 57 |
| 2.5 | PKS-like, NRPS, NRPS-like | 853,548 | 929,386 | friulimicins A-D | 12 |
| 2.6 | PRE-containg | 1.122.831 | 1,145,083 |  |  |
| 2.7 | NAPAA | 1,175,415 | 1,209,299 |  |  |
| 3.1 | transAT-PKS, T1PKS, hgIE-KS | 133,657 | 205,822 | rifamorpholines A-E | 11 |
| 3.2 | T3PKS, Redox-cofactor | 353,742 | 440,721 | julichromes Q3-3, Q3-5 | 37 |
| 3.3 | T3PKS | 1,006,491 | 1,047,651 |  |  |
| 4.1 | Ectoine | 816,967 | 827,353 | ectoine | 100 |
| 5.1 | Lanthipeptide III | 382,776 | 405,406 | ery3-9 | 75 |
| 6.1 | indole | 356,114 | 377,097 | frankiamicin | 14 |
| 6.2 | Ectoine | 671,017 | 681,388 | kosinostatin | 6 |
| 6.3 | NRPS-like, T1PKS, PRE-containing | 687,011 | 766,413 | amycolamycins A,B | 45 |
| 7.1 | terpene | 112,485 | 133,411 | isorenieratene | 42 |
| 7.2 | RiPP-like | 566,684 | 577,484 |  |  |
| 10.1 | T1PKS, Butyrolactone | 67,142 | 171,081 | macrotermycins | 96 |
| 10.2 | terpene | 241,393 | 263,417 | geosmin | 100 |
| 11.1 | PKS-like | 258,312 | 299,421 |  |  |
| 14.1 | terpene | 14,321 | 35,256 | isorenieratene | 71 |
| 14.2 | NRPS, NRPS-like | 89,220 | 135,474 | limazepines A, C-F | 100 |
| 15.1 | NRPS | 139,148 | 211,464 | dechlorocuracomycin | 12 |
| 16.1 | NRPS | 1 | 46,518 | scabechelin | 80 |
| 18.1 | PKS-like, Lanthipeptide I | 36,396 | 79,511 | A5145 | 3 |

NRP - non-ribosomal peptide, RiPP-like - other unspecified ribosomally synthesised and post-translationally modified peptide product (RiPP) cluster, NAPAA - non-alpha poly-amino acids like e-Polylysin,

# Fig. S8 The genetic map of the macrotermycin (Mte) biosynthetic gene cluster:

in genome of *Amycolatopsis* sp. M39 (Beemelmanns *et al.*, 2017), *Amycolatopsis* A23^T^ (B) and *Amycolatopsis pretoriensis* DSM 44654^T^ (C).


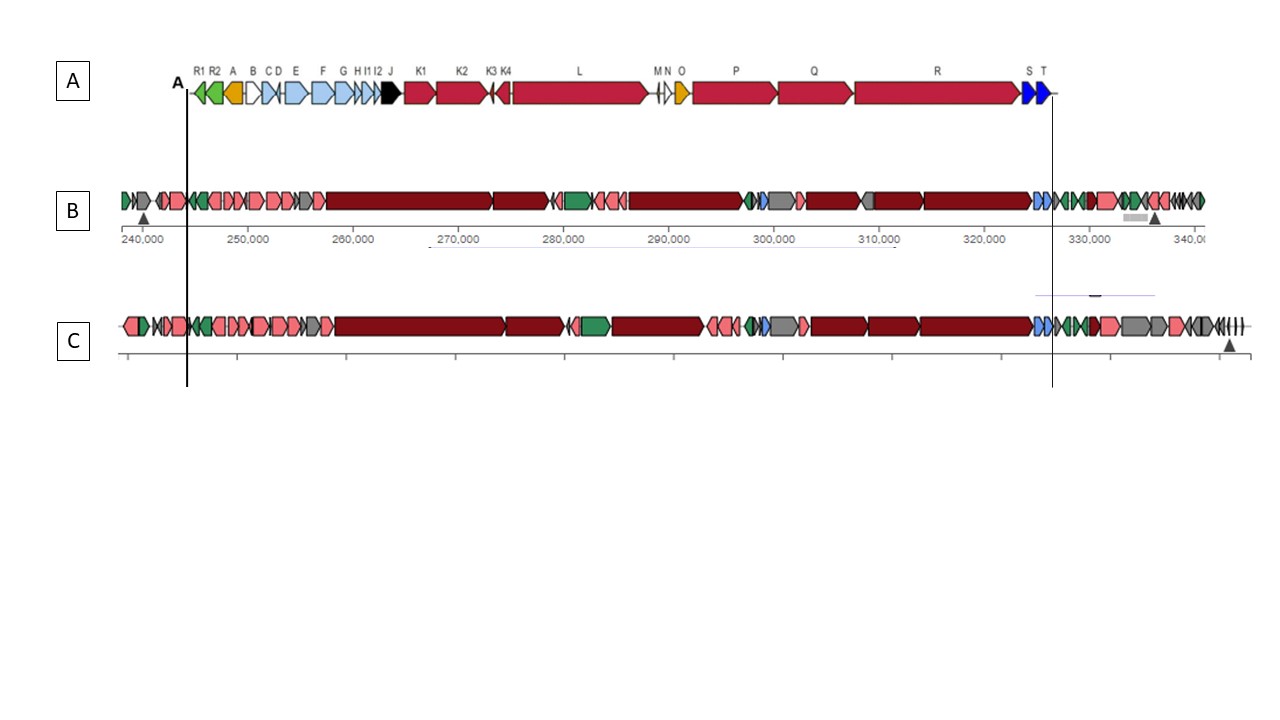


# Table S7. Probability of being a human pathogen: family and function of pathogenic proteins in *Amycolatopsis* genomes

| Strains | Probability of being a human pathogen | Matched Pathogenic Families | Organisms | Protein Function | Protein Id | % Identity |
| --- | --- | --- | --- | --- | --- | --- |
| *A. australiensis* DSM 44671^T^ | 0.352 | 2 | *Gordonia bronchialis* DSM 43247, complete genome. | methane/phenol/toluene hydroxylase | ACY22743 | 90.09 |
|  |  |  | *Saccharomonospora viridis* DSM 43017 | ABC-type transport system involved in resistance to organic solvents, permease component | [ACU95447](http://www.ncbi.nlm.nih.gov/protein/ACU95447.1) | 88.4 |
| *A. pretoriensis* DSM 44654^T^ | 0.258 | 1 |  |  |  | 88.4 |
| *Amycolatopsis sp.* A23 | 0.26 |  |  |  |  | 88.4 |
| *A. balhimycina* DSM 44591T | 0.252 |  |  |  |  | 88 |
| *A. eburnea* NBRC 113658T | 0.242 |  |  |  |  | 88 |
| *A. kentuckyensis* NRRL B-24129T | 0.247 |  |  |  |  | 89.2 |
| *A. lexingtonensis* NRRL B-24131^T^ | 0.266 |  |  |  |  | 88.8 |
| *A. mediterranei* NRRL B-3240^T^ | 0.28 |  |  |  |  | 88.8 |
| *A. rifamycinica* DSM 46095^T^ | 0.27 |  |  |  |  | 88.8 |
| *A. tolypomycina* DSM 44544^T^ | 0.26 |  |  |  |  | 89.2 |
| *A. vancoresmycina* DSM 44592^T^ | 0.318 | 2 |  |  |  | 89.2 |
|  |  |  | *Nocardia farcinica* IFM 10152 DNA | putative aliphatic amidase | [BAD55601](http://www.ncbi.nlm.nih.gov/protein/BAD55601.1) | 87.54 |
| *A. vastitatis* NRRL B-65279^T^ | 0.263 | 1 | *Saccharomonospora viridis* DSM 43017 | ABC-type transport system involved in resistance to organic solvents, permease component | [ACU95447](http://www.ncbi.nlm.nih.gov/protein/ACU95447.1) | 88.8 |
